# UMOT: A unified framework for long- and short-term association for multi-object tracking

**DOI:** 10.1371/journal.pone.0332709

**Published:** 2025-09-26

**Authors:** Yinghong Xie, Yongxing Ke, Xiaowei Han, Qiang Gao, Chongli Wang

**Affiliations:** School of Intelligent Science and Information Engineering, Shenyang University, Shenyang, Liaoning, China; Centro de Investigacion en Ciencias de Informacion Geoespacial AC (Research Center on Geospatial Information Sciences), MEXICO

## Abstract

Multi-object tracking (MOT) is an important research direction in the field of computer vision, but it often leads to problems such as trajectory breakage and identity switches in complex scenarios due to the similarity of target appearance and long-term occlusions. Although the existing Transformer-based end-to-end methods have made some breakthroughs in short-term motion modelling, they are still deficient in long-term dependent information modelling and target recovery. To this end, this paper proposes a unified framework for long-term and short-term association, UMOT, which fundamentally alleviates the contradiction between motion prediction between neighboring frames and long-term trajectory recovery across frames. UMOT constructs short-term correlation using pre-trained YOLOX detector and MOTR-ConvNext network, and through the dynamically updated track queries and detect queries jointly to build the motion and appearance feature models of targets between adjacent frames to optimize the short-term motion prediction. Meanwhile, it effectively stores and smoothly updates the historical trajectory information by designing the Track Query Memory Module (TQMM) and associates the unmatched detections across frames by the Historical backtracking Module, so as to realize the restoration of the long-term lost targets and the identity preservation. Extensive experiments on DanceTrack and MOT17 datasets show that UMOT achieves significant improvement in HOTA, IDF1 and other metrics compared to existing state-of-the-art methods, which verifies its robustness and effectiveness in complex occlusion and long-term dependency scenarios.

## Introduction

The core challenge of multi-object tracking (MOT), which aims to continuously locate and associate multiple targets in a video sequence, lies in how to effectively model the spatio-temporal dynamics in complex scenarios. Although the Tracking-by-Detection (TBD) tracking paradigm has made significant progress by separating the detection and association tasks (e.g., SORT [1], DeepSORT [2]), its reliance on the design of heuristic matching rules (e.g., IoU, Re-ID features) and post-processing strategies (e.g., trajectory interpolation) leads to a broken spatio-temporal information flow that it is difficult to cope with long-term occlusions, target disappearance and identity switches in complex motion scenarios. In recent years, Transformer-based end-to-end methods (e.g., MOTR [3], MOTRv2 [4]) have significantly improved the robustness of short-term associations by iteratively updating the track queries to implicitly model target motion and appearance changes.

However, there are still two major bottlenecks in such methods. The first is the optimization conflict between the detection and tracking tasks. MOTR [3] jointly optimizes the detect queries with the track queries, but the detection performance is limited by Transformer’s end-to-end framework, which leads to high leakage and false detection rates. MOTRv2 [4], though, by introducing a pre-trained detector (e.g., YOLOX [5]) to generate the proposal queries alleviates the detection problem, the implicit decoupling of detection and tracking still can not completely eliminate the gradient interference between tasks. Secondly, long-term dependency modelling is insufficient; existing methods mainly rely on local information of adjacent frames and lack global awareness of multi-frame historical context. For example, MOTR achieves short-term trajectory information retention by fusing the track queries of the current and previous frames through a temporal aggregation network (TAN), but its online processing mechanism and exit strategy limit the ability of trajectory recall for long-term occluded or disappeared targets. Although MOTRv2 provides positional priors through the detector, it is not designed with a recovery mechanism for long-term lost targets, which makes it difficult to solve the problem of trajectory breakage in occlusion scenarios.

To address the above challenges, this paper proposes a unified framework for multi-object tracking (UMOT) that jointly models short-term motion prediction and long-term trajectory recovery within an end-to-end architecture. The proposed approach aims to enhance identity consistency and tracking robustness in complex scenarios involving frequent occlusions and reappearances. The main contributions of this work are summarized as follows:

(1) We propose a short-term correlation module that couples a pre-trained YOLOX detector with the MOTR-ConvNeXt network. By leveraging dynamically updated track queries and detect queries, this module accurately models motion and appearance transitions between adjacent frames to optimize short-term motion prediction. The use of the ConvNeXt backbone [6] with large receptive fields and hierarchical features improves the contextual perception of occluded or overlapping targets, thereby enhancing short-term association stability.
(2) We develop a long-term correlation module composed of a Track Query Memory Module (TQMM) and a Historical backtracking Module (Hb). The TQMM persistently stores multi-frame trajectory features and maintains temporal continuity via Temporal and Spatial Enhancement Module (TEM) and Exponential Moving Average (EMA) strategies. The Hb module performs global cross-frame correlation between unmatched detections and memory trajectories, enabling effective recovery of long-term lost targets and significantly reducing identity switches.
(3) We integrate the above modules into a unified framework that supports joint short- and long-term association in an end-to-end learnable manner. Extensive experiments on DanceTrack and MOT17 benchmarks demonstrate that UMOT significantly improves metrics such as IDF1 and HOTA, achieving a HOTA gain of 0.9% on MOT17 compared to MOTRv2 and an IDF1 gain of 2.1% on DanceTrack compared to MOTRv2, thereby confirming its robustness in identity preservation and long-term trajectory continuity.

## Related works

Traditional MOT methods follow a detection-association two-stage paradigm, e.g., SORT [1] predicts the target location by Kalman filtering [7] and uses the Hungarian algorithm [8] for IoU matching, and DeepSORT [2] further introduces Re-ID features to calculate the appearance similarity. FairMOT [9] improves feature consistency by jointly training the detection and Re-ID branches, while ByteTrack [10] improves tracking robustness by optimizing the correlation strategy of detection frames. And OC-SORT [11] enhances robustness to complex nonlinear motions through observation-centred error correction and momentum-consistent design to improve association accuracy. However, such methods rely on post-processing and matching strategies, resulting in the inability to jointly optimize the detection and association tasks and difficulty in modelling long-term temporal dependencies.

In recent years, Transformer-based end-to-end methods [12] have made breakthroughs by implicitly modelling spatio-temporal relationships. MOTR [3] extends the detect queries of DETR [13] into a track queries, which achieves cross-frame tracking through iterative updating. However, its detection performance is limited by optimization conflicts in the end-to-end framework. MOTRv2 [4] introduces pre-trained detector to generate proposal queries, implicitly decouples the detection and tracking tasks, which significantly improves the detection accuracy, but still lacks a recovery mechanism for long-term lost targets. MOTRv3 [14] gradually adjusts the label assignment process through the RFS strategy, so that the detect queries can fully participate in the training, and thus solves the optimization conflict between detection and tracking more efficiently. However, it can only solve the correlation on a short period of time well, and it is still difficult to address associations over long periods of time with long-term occlusions or loss. TransTrack [15] associates historical trajectories with current detections through cross-attention, but relies on hand-crafted matching strategies (e.g., IoU thresholds) and fails to achieve global contextual awareness. TrackFormer [16] associates historical trajectories with current detections through cross-attention and uses the self-attention mechanism to implicitly model the global dependencies of targets in spatio-temporal context, but still not completely free from manual rules.

For the long-term occlusions problem, MeMOT [17] stores target features by constructing short-term and long-term memory banks, and deeply integrates them into the query process of detection and association through memory encoding and decoding mechanisms. GTR [18] adopts a memory bank but does not fully incorporate a dynamic query update mechanism. TransMOT [19] models cross-frame associations using a spatio-temporal Transformer, but its computational complexity rises drastically with the growth of video length. In contrast, UMOT proposes the Track Queries Memory Module (TQMM), which dynamically aggregates multi-frame track queries through the Temporal Enhancement Mechanism (TEM) and smoothly updates the history state in conjunction with the Exponential Moving Average (EMA) strategy [20], which achieves the long-term dependency modelling while guaranteeing computational efficiency. In addition, the History backtracking Module performs global correlation analysis between unmatched detections and lost memory tracks through the cross-attention mechanism, which significantly reduces the false correlation rate under complex motion.

For feature extraction, most of the existing methods use ResNet or Swin Transformer to extract features, the local receptive field of ResNet [21] limits the contextual awareness, while the high computational cost of Swin Transformer [22] is difficult to meet the real-time demand. UMOT introduces ConvNeXt [6] as the backbone network with depthwise separable convolution with inverted bottleneck structure expands the receptive field while maintaining lightweight, and enhances the localization and discrimination of occluded targets through multi-scale feature fusion.

Based on the above analyses, this paper focuses on exploiting temporal information and proposes UMOT, a unified framework for long-term and short-term association for multi-object tracking. UMOT aims to effectively solve the problem of tracking failures as well as the problem of mis-matching of targets after they have been lost over a long or short period of time. Specifically, UMOT achieves this goal by performing two steps of short-term association and long-term association for each video frame. In short-term association, the bounding boxes of frame t-1 and frame t are first obtained by the YOLOX detector, and then proposal queries are generated by borrowing from MOTRv2, which are used to replace the detect queries in MOTR-ConvNext to detect the nascent targets. MOTR-ConvNext is a network model that replaces the Resnet network in MOTR by the ConvNext network. The track queries of frame t-1 and detect queries of frame t-1 are then spliced and fed into the decoder in MOTR-ConvNext along with the image features to predict the bounding box of frame t and perform motion prediction of the tracked target, followed by IOU matching of the predicted and detected boxes of frame t to achieve the short-term association. Long-term association then involves the computation of the correlation between the detect queries corresponding to the unmatched target and the lost memory track queries stored in the TQMM module to recover targets that have disappeared over a long period of time. Finally, UMOT combines the results of short-term and long-term correlations to generate complete tracking results.

In summary, UMOT achieves a balance between collaborative optimization of detection and tracking tasks and long-term dependency modelling in multi-object tracking by means of a unified framework for long- and short-term correlation, providing a new solution for multi-object tracking in complex scenarios.

## Method

### Overall architecture

In this work, we propose a unified framework for multi-object tracking (UMOT) that integrates a short-term correlation module and a long-term correlation module. This architecture enables the model to simultaneously perform short-range motion estimation and long-range identity association within a single end-to-end trainable framework.

The short-term correlation module is built upon the combination of a YOLOX detector and a MOTR-ConvNeXt network and is designed to capture appearance and motion transitions between adjacent frames. By fusing track queries and detect queries, the module learns discriminative spatio-temporal embeddings that guide accurate bounding box regression and identity association for both ongoing and newly emerged targets. The long-term correlation module addresses target loss caused by extended occlusions or missed detections. It consists of two key components: the Track Query Memory Module (TQMM) and the Historical Backtracking Module (Hb). The TQMM continuously stores and updates long-term trajectory features using the Temporal and Spatial Enhancement Module (TEM) and Exponential Moving Average (EMA) strategies. The Hb module performs cross-attention-based correlation between unmatched detections and memory-stored track queries. This enables reliable identity reassociation and trajectory recovery across long temporal gaps.

By jointly modeling short-term and long-term associations, the UMOT framework effectively balances short-term localization precision with long-term identity continuity. As a result, it demonstrates strong robustness across a wide range of tracking scenarios, including crowded scenes, frequent occlusions, complex motion patterns, and high appearance similarity.

**Fig 1:**
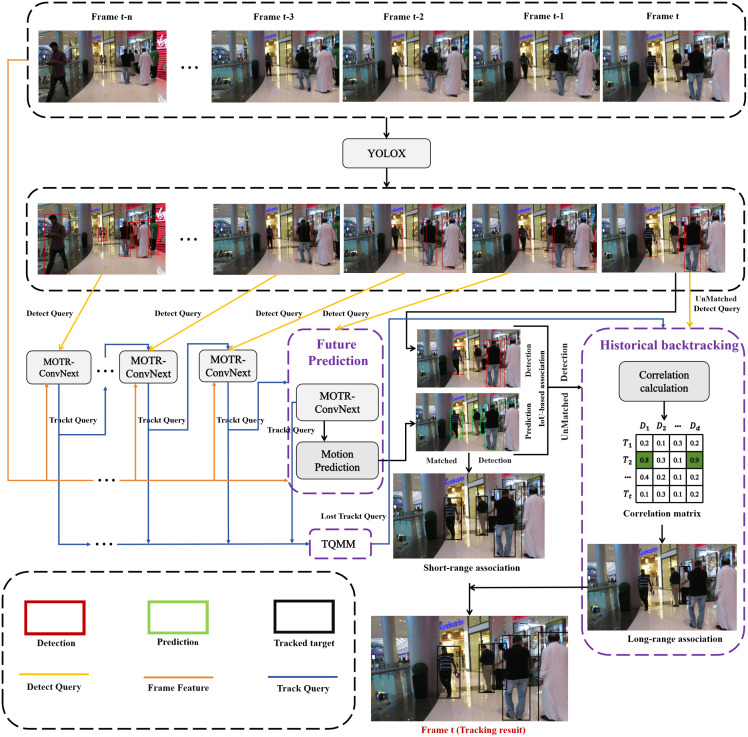
The overall architecture of UMOT.

UMOT mainly performs two steps of short-term association and long-term association for each video frame. The overall structure is shown in Fig 1.

Short-term association, given an input of *t* frames of video, this method first obtains the detection result $D_{t-1}\in R^{W_{t-1}\times 5}$ of frame *t*-1 and the detection resul*t* $D_{t}\in R^{W_{t-1}\times 5}$ of frame *t* through the YOLOX detector, and the detection resul*t* of each frame is represented by W bounding boxes, and each bounding box is represented by the centre coordinates (x,y), the height h, the width w and the confidence level s. The bounding box is then used to generate a set of proposal queries by introducing a shared query, which replaces the detect queries $Q_{d}\in R^{L\times D}$ in MOTR-ConvNext to detect the newborn targets, L is the number of newborn targets, and D is the dimension of each trajectory. Meanwhile, this method obtains the track queries $Q_{tr}^{t-1}\in R^{N_{t-1}\times D}$ of frame *t*-1 ($N_{t-1}$ is the number of trajectories in frame *t*-1) from the MOTR-ConvNext of frame *t*-2, and then concatenates the track queries $Q_{tr}^{t-1}$ of frame *t*-1 together with the detect queries $Q_{d}^{t-1}\in R^{L_{t-1}\times D}$ of frame *t*-1 ($L_{t-1}$ is the number of newborn targets in frame *t*-1). The spliced queries and the image features $f_{t-1}$ of frame *t*-1 into the decoder in MOTR-ConvNext, where these queries interact with the image features to generate the hidden state, which in turn predicts *t*he bounding box of the tracked object at frame *t*. Motion prediction is performed for *t*he tracked targe*t*, and the hidden state is also fed back to the Query Interaction Module (QIM) in the MOTR-ConvNext model *t*o generate the track queries $Q_{tr}^{t}$ for the next frame (frame *t*). Meanwhile, the track queries are stored in the TQMM module *t*o provide up-to-date trajectory information of the lost *t*arget for long-term association. Finally, the prediction boxes of the frame *t* are matched with the IOU of the detection boxes of the frame *t*, and the short-term association is realized with the goal of successful matching.

For long term association, assuming that there are d unmatched detections after the short term association of frame *t*, the correlation is calculated be*t*ween its corresponding detect queries $Q_{dtu}\in R^{d\times D}$ and memory track queries $Q_{trml}\in R^{t\times D}$ in the lost state stored in the TQMM module (assuming that there are *t* memory tracks in the lost state stored in the TQMM). The correlation matrix $A^{sim}\in {\left[0,1\right]}^{t\times d}$ is obtained through *t*he History backtracking Module, where each element indicates the association probability of an unmatched detection target to another memory tracks in the lost state, i.e., the two queries with high correlation correspond to the same target, and the successful match is the one that achieves long-term association. If there is still no successful match, i.e., the correlation between the detect queries of the unmatched detection target and the track queries of all the targets in the lost state stored in the memory module are all low, the detection target is considered as a new target, which is used for target prediction in the next frame and generates the track queries of the new target.

Finally, we combine the results of short-range and long-range correlations to generate the complete tracking results for frame *t*. The unification of short-range and long-range associations for target tracking is thus achieved.

### Future forecasting module (MOTR-ConvNext)

In this paper, MOTR-ConvNext is used to predict the motion information of the tracked target in the future frames, the motion information includes centre coordinates (x,y), height h, widthw, confidence level s and appearance information.

MOTR performs the multi-object tracking task by iteratively predicting a sequence of target trajectories. It extends object queries in DETR to track queries, which act as hidden states of the tracked instances and are passed and updated across frames for timing modelling. The sequence of hidden states of each track queries correspond to the trajectory of a target, and its hidden states are iteratively updated in the Transformer decoder through a self-attention and cross-attention mechanism, and are used to predict the bounding box frame by frame. The update process of the track queries implicitly jointly learns the appearance and motion characteristics of the target, while end-to-end tracking is achieved through dynamic management mechanisms (e.g., the addition of newborn queries and the removal of disappearing queries) without post-processing matching. In addition, Tracklet-Aware label assignment ensures that each track queries is strictly aligned with the target sequence of the same identity, while Temporal Aggregation Network (TAN) and Collective Averaging Loss (CAL) further enhance the temporal modelling capability through historical information aggregation and whole video supervision, respectively.

Despite the better short-term association of MOTR, the detection results are not as good as those of the tracking-by-detection methods. Therefore, this paper draws on the idea of MOTRv2 to guide end-to-end multi-object tracking using pre-trained target detectors, i.e., by combining the YOLOX target detector to generate proposals that act as object anchors to provide detection priors to MOTR, providing stable positional priors for the track queries initialization, which in turn improves the MOTR. Meanwhile, in order for the backbone network to have better feature representation capability, better long-distance dependency modelling and higher efficiency, this paper adopts ConvNeXt network instead of the original ResNet backbone network to construct the MOTR-ConvNeXt model.

**Fig 2:**
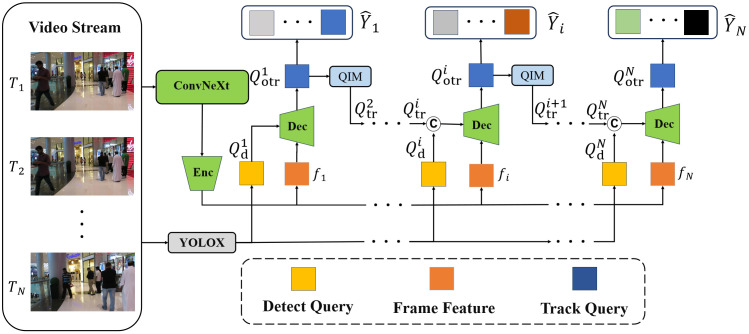
Structure of MOTR-ConvNext.

The cited benefits of ConvNeXt network [6] are mainly reflected in the following aspects. First, ConvNeXt adopts 7 × 7 depth-separable convolution, which significantly enlarges the receptive field compared to the 3 × 3 convolution of ResNet. In the occlusion scene, the larger receptive field can capture the contextual information of the target’s surroundings, assist in inferring the location of the occluded part, and improve the perception of local details. Second, through the design paradigm of inverted bottleneck structure, ConvNeXt optimizes the transfer efficiency of features, reduces the loss of information, and reduces the phenomenon of feature degradation during gradient updates, which is crucial for maintaining the identity continuity of occluded targets. Third, ConvNeXt simplifies the network complexity by streamlining the network components and retaining only the necessary LayerNorm and GELU activations, focusing the feature representation on key semantic regions and effectively suppressing occlusion-induced noise interference. ConvNeXt’s multi-stage design generates feature maps with different resolutions, with low-resolution features capturing the global context and high-resolution features preserving the local details. Such hierarchical features complement MOTR’s Transformer decoder to enhance the localization and identity association of occluded targets. ConvNeXt is naturally adapted to MOTR’s end-to-end framework as a purely convolutional network without the need to adjust the input resolution or positional encoding. Its efficient computational properties (e.g., high throughput) enhance tracking real-time. The structure of MOTR-ConvNext is shown in Fig 2.

As shown above, the whole model iteratively processes each video frame. The video sequence is first fed into the ConvNext backbone network (e.g., ConvNext-Base) and the Deformable DETR [23] encoder extracts image features $f=\left\{f_{1},...,f_{i},...,f_{N}\right\}$, here $f_{1}$ denotes as features of frame $T_{1}$, while the video sequence passes through the YOLOX detector to obtain the detect queries $Q_{d}=\left\{Q_{d}^{1},...,Q_{d}^{i},...,Q_{d}^{N}\right\}$ for detecting the nascent target. The QIM generates the track queries $Q_{tr}^{i}(i\in [2,N])$ based on the initial track queries $Q_{otr}^{i-1}$ of the previous frame ($Q_{tr}^{1}$ is generated by the initialization), $Q_{tr}^{i}$ is cascaded with the corresponding detect queries of each frame, and the cascaded queries and features $f_{i}(i\in [1,N])$ are fed into the Decoder for interaction to generate the initial track queries $Q_{otr}^{i}$ of the current frame. The initial track queries $Q_{otr}^{i}$ is hidden, and its function is to predict the bounding box of the tracked object in the next frame, and to predict the motion of the tracked target. The prediction result for the current frame is then generated through $Q_{otr}^{i}$, while the initial track queries is fed back into the Query Interaction Module (QIM), which is then updated through the QIM to generate $Q_{tr}^{i+1}$ to feed it into the next frame. For frame $T_{1}$ (first frame), the track queries is initialized to be empty, and only the feature $f_{1}$ and detection queries $Q_{d}^{1}$ are fed into the Decoder for interaction and generation of the initial track queries $Q_{otr}^{\text{1}}$ for the first frame, which is followed by the generation of the prediction ${\hat{Y}}_{1}$ for the current frame as well as the track queries $Q_{tr}^{\text{2}}$ for the next frame (frame $T_{2}$).

### Track queries memory module (TQMM)

**Fig 3:**
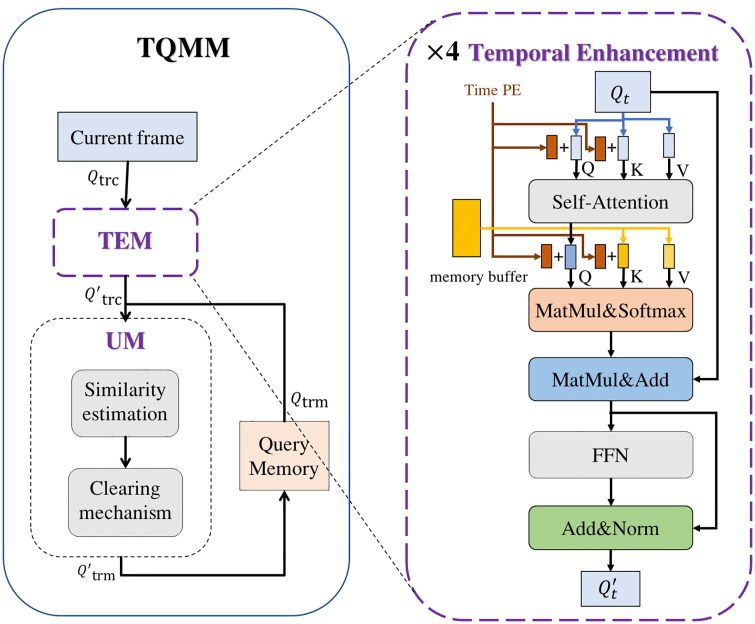
Structure of TQMM module.

In the above description, QIM is responsible for accepting the initial track queries for the current frame and generating the track queries for the next frame, while the track queries generated whenever the previous frame is generated is also stored in the TQMM module, which provides the latest trajectory information of the lost target for the subsequent long-term correlation. The TQMM module is shown in the following Fig 3.

As shown in the Fig 3, the current frame gets the track queries $Q_{trc}$ of the current frame through the MOTR-ConvNext of the previous frame, and then $Q_{trc}$ passes through the Temporal and Spatial Enhancement Module (TEM) to get the track queries ${Q^{\prime }}_{trc}$ of the current frame fused with the context information, and the track queries of each frame includes the trajectory information of multiple targets, in which the trajectory information includes the number of frames, the ID of the target, the target’s appearance and the position information. ${Q^{\prime }}_{trc}$ is estimated for similarity with the memory track queries stored in the Query Memory Module, where the Query Memory Module is responsible for storing the track queries information of all the targets that have appeared, and then the trajectory information in the TQMM is continuously updated by means of a storage mechanism and a clearing mechanism set up in the UM (Update Module). Since the track queries is initialized as empty, the query memory module replaces the track queries $Q_{tr}^{\text{1}}$ of the first frame with the detect query $Q_{d}^{\text{1}}$ generated from the first frame of the picture to be deposited into the query memory module as the initial information to be stored, and at the same time initializes the un-updated counter of the target in $Q_{tr}^{\text{1}}$ (set to 0).

#### Temporal and spatial enhancement module (TEM).

TQMM fuses information from track queries between multiple historical frames and the current frame by establishing TEM, and TEM integrates the information of historical frames and the current frame through the attention mechanism, and obtains the track queries of the current frame which integrates the context information.

Memory buffer, in this paper, we set up a memory buffer to store the information of track queries of multiple history frames in TEM, the buffer can store maximum $T_{S}$ frames of time length, and each frame can save the information of at most M objects. This buffer is implemented using a First-In-First-Out (FIFO) data structure, where the oldest data in the queue is discarded as new frame information is added. The FIFO mechanism ensures that the memory consumption of the system remains constant, even in the case of constant integration of new information, making it highly suitable for environments with limited computational resources.

TEM, TEM enhances the information of the current frame by aggregating the track queries between the previous $T_{S}$ frames and the current frame, which is implemented as follows:

Assume that the dimension of each query in each frame is D, with frame *t* as the current frame, $N_{t}$ track queries in frame *t*, and $N_{t-T_{s}},N_{t-T_{s}+1}\cdot \cdot \cdot N_{t-1}$ track queries in the previous $T_{S}$ frames (history frames). Respectively, $Q_{t}\in R^{N_{t}\times D}$ for the current frame, and $Q_{t-T_{s}}\in R^{N_{t-T_{s}}\times D},Q_{t-T_{s}+1}\in R^{N_{t-T_{s}+1}\times D}\cdot \cdot \cdot Q_{t}\in R^{N_{t}\times D}$ for the previous $T_{S}$ frames (his*t*ory frames). We use padding and masking to ensure that the size of the input tensor is the same for each point in time, as well as to provide information to ignore invalid padding data.

As shown in Fig 3, TEM consists of four layers, each consisting of four main components: a self-attention layer, a matrix multiplication and Softmax layer, a weighted summation layer, and a feed-forward neural network layer. We convert timestamps into location embeddings to further refine the processing power of the model. Queries stored in the buffer represent historical data, providing a rich contextual background that significantly enhances the model’s understanding of temporal dynamics. The track queries $Q_{t}\in R^{N_{t}\times D}$ of the current frame is first updated by self-attention and then used as Q by linear transformation, while the historical track queries in the memory buffer acts as both K and V by linear transformation:


$$\left\{ \;\;\;\begin{array}{c} Q=W_{q}(Q+P^{t})\in R^{N\times D}\\ K=W_{k}(Q_{trc}^{h}+P^{h})\in R^{N\times D}\\ V=W_{v}Q_{trc}^{h}\in R^{N\times D} \end{array}\right.$$ (1)

Where $P^{t}$ and $P^{h}$ are time coding matrices, $W_{q}$, $W_{k}$ and $W_{v}$ are linear transformation matrices, and N is the number of trajectories and the maximum number of targets in $T_{S}+1$ frames. For frames with number of targets less than N, they are filled with zero vectors up to N.

In the following, we perform a matrix product of Q and K and then go through the Softmax layer to get the similarity matrix:


$$A=softmax(\frac{QK^{T}}{\sqrt{d_{k}}}+M)\in R^{N\times N}$$ (2)

Where $d_{k}$ is the dimension of the key vector, $QK^{T}$ denotes the dot product between the query and the key, this step is used to measure the similarity between the query and all the keys, after which it is divided by $\sqrt{d_{k}}$ to scale the result of the dot product, where $M\in R^{N\times N}$ is the mask matrix, which is used to ignore the effect of filler items. In the following, the similarity matrix is used to weight the historical track queries, the similarity matrix A is multiplied by V to get the historical track queries that incorporates the information from the current frame, i.e., softmax(QKT/QKTdk\nulldelimiterspacedk+M)V∈RN×D, and then the current track queries is summed up with the enhanced historical track queries to generate the current track queries that incorporates the information from the historical frame:


$${\hat{Q}}_{t}=softmax(\frac{QK^{T}}{\sqrt{d_{k}}}+M)V+Q_{trc}^{t}\in R^{N\times D}$$ (3)

Finally, the feed-forward network [24] (FFN) is utilized for further tuning, and finally the track queries $Q_{t}^{\prime }$ with fused contextual information at frame t is obtained:


$$Q_{t}^{\prime }=LN(FC(Re(FC({\hat{Q}}_{t})))+{\hat{Q}}_{t})\in R^{N\times D}$$ (4)

where FC denotes linear projection layer, LN denotes layer normalization and Re denotes ReLU activation function.

#### Update module (UM).

TQMM estimates the similarity between the track queries of the current frame fused with contextual information and the memory track queries $Q_{trm}$ stored in the query memory module through UM, then stores the information of the current track queries through the set storage mechanism and similarity, and at the same time clears the track queries that has not been updated for a long period of time through the clearing mechanism, which in turn continuously updates the trajectory information in TQMM.

Query Memory Module, as shown in Fig 3, we store the historical track queries of all the tracked objects that have appeared in the Query Memory Module. It maintains the maximum length of the memory bank through a sliding window mechanism with a window length of $T_{w}$ frames, which stores information about at most M objects.

Similarity estimation, as it is necessary to estimate the similarity between the memory track queries $Q_{trm}$ in the Query Memory Module and the track queries $Q_{trc}^{\prime }$ of the current frame that incorporates contextual information, this paper allows each trajectory in the current track queries to match each trajectory in the memory track queries through cosine similarity to obtain the similarity score. Cosine similarity is a measure of the angular difference between two vectors, which is independent of the length of the vectors and is well suited for calculating the similarity between high-dimensional feature vectors. When calculating cosine similarity, we first perform L2 normalization of the eigenvectors and then perform matrix multiplication. This ensures that the cosine similarity is computed and not a normal dot product. The cosine similarity formula is as follows:


$$S_{ij}=cosine\_similarity(c_{i},m_{j})=\frac{c_{i}\cdot m_{j}}{\left\|c_{i}\right\|\left\|m_{j}\right\|}$$ (5)

Where $S_{ij}$ denotes the cosine similarity between the *i* th trajectory $c_{i}$ in track queries $Q_{trc}^{\prime }\in R^{N\times D}$ of the current frame and the *j* th trajectory $m_{j}$ in memory track queries $Q_{trm}\in R^{M\times D}$. M and N are the number of trajectories, and D is the vector dimension.

Calculating the cosine similarity needs to involve a loop traversing the entries of each track query and memory track query, which is not efficient. Therefore, matrix operations are used to calculate the similarity between all the entries of the track query and the memory track query in one go, which improves the speed of computation. A similarity matrix $S\in R^{N\times M}$ is finally obtained, and each element of this matrix represents the degree of similarity of the motion between two trajectories.

The query memory module is updated, then the similarity threshold S∈[0,1] is set to determine whether the target in the current frame is a new target, and then the memory track queries in the query memory module is updated according to the update mechanism. If the similarity of the target in the current frame is greater than or equal to the similarity threshold S, then the target is an old target (one that has already been tracked), and the trajectory information of the target is smoothed to overwrite the corresponding information in the memory track queries, and at the same time, an un-updated counter is initialized (counting the number of frames in which the target information has not been updated). Since direct overlay may lead to abrupt changes in feature information, we use exponential moving average [20] (EMA) to smooth information updates:


$$EMA_{t}=\alpha x_{t}+(1-\alpha )EMA_{t-1}$$ (6)

Where $EMA_{t}$ is the exponential moving average at time *t*, $x_{t}$ is the actual data value a*t* time *t*, and $EMA_{t-1}$ is the exponential moving average at time *t*-1. α is the smoothing fac*t*or hyperparameter, usually α∈(0,1).

If the similarity of the target in the current frame is less than the similarity threshold S, the target is a new target (newly appeared target), and the corresponding trajectory information of the target is stored in the Query Memory Module, and at the same time the un-updated counter is initialized.

Memory clearing mechanism, the track queries in the Query Memory Module is divided into three states: alive, lost and inactivated, and the frame threshold $F\in (1,T_{W})$ is set through the clearing mechanism to judge the state of the memory track queries in the Query Memory Module. The un-updated counter is updated frame by frame, if the value of the un-updated counter of a target in the memory track queries is 0, the target is alive and recorded as $q_{trma}$, if the value of the un-updated counter of a target in the memory track queries is greater than 1 and less than or equal to F, the target is lost and recorded as $q_{trml}$. If the value of the un-updated counter of a target in the memory track queries is greater than F, the target is inactivated and recorded as $q_{trmi}$ and the Query Memory Module deletes the trajectory information of the target.

### Historical backtracking module

**Fig 4:**
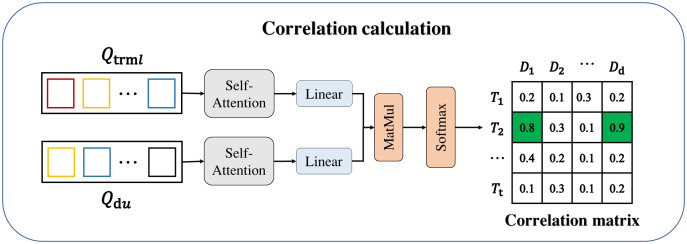
Structure of Historical backtracking Module.

Through the History backtracking Module, the detect queries of the unsuccessfully matched detection targets in the short-term correlation is correlated with the memory track queries stored in the TQMM module in the lost state, and the correlation magnitude between the targets of the two track queries is used to judge whether the two targets are the same target. The structure of History backtracking Module is shown in Fig 4.

As shown in the Fig 4, the detect queries $Q_{du}\in R^{W\times L}$ of unmatched targets and the memory track queries $Q_{trml}\in R^{M\times D}$ in the lost state are put through self-attention, linear transformation, matrix proportionality and Softmax layer to get the correlation between the two queries. Firstly, the two queries capture the dependencies within the sequences through an independent self-attention mechanism to generate an enhanced feature representation, the self-attention formula is as follows:


$$Self-Attention(Q,K,V)=softmax(\frac{QK^{T}}{\sqrt{d_{k}}})V$$ (7)

Where Q, K, and V are the Query, Key, and Value matrices respectively. $d_{k}$ is the dimension of the key that is used to scale the attention weights. We then implement the computation of relevance by borrowing from the cross-attention approach, so that each element in one sequence can pay attention to all the elements of the other sequence and get this level of attention. We still use padding and masking [25] to ensure that the two input tensors are of the same size, while providing information to ignore invalid padding data. Two track queries ${\hat{Q}}_{trml}\in R^{M\times D}$, ${\hat{Q}}_{du}\in R^{M\times D}$ of the same size are obtained by padding, M is the number of trajectories after zero-vector padding and the maximum number of targets in the two track queries (assuming that there are the maximum number of targets in the memory track queries), and D is the dimensionality of each query. Then the lost memory track queries ${\hat{Q}}_{trml}$ is linearly transformed to get the query (Q), i.e., $Q=W_{q}{\hat{Q}}_{trml}\in R^{M\times D}$, the detect queries ${\hat{Q}}_{du}$ is linearly transformed to get the key (K), i.e., $K=W_{k}{\hat{Q}}_{du}\in R^{M\times D}$, matrix product of Q and K, and then Softmax function to get the correlation matrix Acor=softmax(QKT/QKTdk\nulldelimiterspacedk+M)∈RM×M, where $M\in R^{M\times M}$ is the mask matrix, which is still used to ignore the effect of the padding term. With the correlation matrix $A^{cor}\in \left[0,1\right]$, we can get the correlation of each trajectory in the detect queries $Q_{du}$ of the unmatched targets to each trajectory in the memory track queries $Q_{trml}$ in the lost state.

### Design of the loss function

In UMOT, in order to achieve effective training for multi-object tracking, we design a comprehensive loss function which takes into account both short-term association and long-term association.

For short-term association, the loss function at frame t is expressed as:


$$L_{t}^{s}=\lambda_{cls}^{s}L_{cls}^{s}+\lambda_{l1}^{s}L_{l1}^{s}+\lambda_{giou}^{s}L_{giou}^{s}$$ (8)

Where $L_{cls}^{s}$ is the classification loss in the short-term association, calculated using focal loss. $L_{l1}^{s}$ is the L1 loss in the short-term association. $L_{giou}^{s}$ is the GIoU loss in the short-term association. $\lambda_{cls}^{s}$, $\lambda_{l1}^{s}$ and $\lambda_{giou}^{s}$ are the corresponding weighting coefficients.

For long-term association, this paper considers the result of correlation calculation between detect queries of unmatched detections and the memory track queries stored in the TQMM module that are in a lost state. The correlation matrix obtained through the History backtracking Module is $A^{cor}\in \left[0,1\right]$. The loss function for long-term association is defined as:


$$L_{t}^{l}=\sum {\mathrm{\backslash nolimits}}_{i=1}^{t}\sum {\mathrm{\backslash nolimits}}_{j=1}^{d}(1-A_{ij}^{cor}{)}^{2}$$ (9)

Where $(1-A_{ij}^{cor}{)}^{2}$ denotes the dissimilarity between the detect queries of unmatched detections and the memory track queries in a lost state, and the loss of long-term association is obtained by summing all dissimilarities.

Finally, UMOT’s total loss function is a weighted sum of short-term association losses and long-term association losses:


$$\;L_{t}=\omega_{s}L_{t}^{s}+\omega_{l}L_{t}^{l}$$ (10)

Where $\omega_{s}$ and $\omega_{l}$ are the weights of short-term association loss and long-term association loss, respectively, used to balance the importance of the two associations in training.

With the loss function design, UMOT can optimize the performance of both short-term association and long-term association during the training process, thus improving the overall effectiveness of multi-object tracking.

## Experiments

### Datasets and metrics

**Fig 5:**
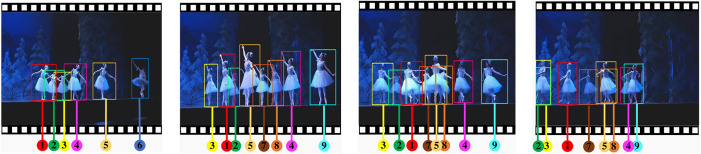
Sample images from the DanceTrack dataset (1^st^, 66^th^, 307^th^ and 327^th^ frame in DanceTrack0027 video).

In order to fully validate the performance of UMOT, we conducted experiments on the following two mainstream multi-object tracking datasets:

1. MOT17: MOT17 (https://motchallenge.net/data/MOT17/) [26] is a widely used dataset containing 7 sequences for training and 7 sequences for testing. It mainly contains relatively crowded street scenes with pedestrians moving in simple straight lines. This dataset is used to verify the generalization ability of the model in regular scenarios.
2. DanceTrack: DanceTrack (https://github.com/DanceTrack/DanceTrack) [27] contains 100 videos of high-density, similar-looking dance scenes with complex motions, each with an average length of 52.9 seconds. This dataset is characterized by a high degree of similarity in target appearance but diverse motion patterns, which makes it suitable for verifying the tracking robustness of the model under prolonged occlusion and complex motion. Sample images from the DanceTrack dataset are shown in Fig 5.

The salient features of this dataset are illustrated in Fig 5: (1) uniform appearance: humans are in highly similar and almost undistinguished appearance. (2) diverse motion: they are in complicated motions and interaction patterns. The numbers below show their identifications which experience frequent relative position switches and occlusions.

The evaluation metrics used for the aforementioned dataset include CLEAR MOT Metrics [28] and HOTA [29]. Specifically, CLEAR-MOT Metrics include IDF1 score [30] (IDF1), multi-object tracking accuracy (MOTA) and identity switches (IDS). Considering that MOTA focuses on detection and short-term tracking, which will ignore trajectory continuity, while the study in this paper focuses on identification, retention, and long-term tracking of target identities with strong trajectory continuity, HOTA is introduced in this paper. HOTA (Higher Order Tracking Accuracy), by balancing the detection accuracy and data association accuracy, overcomes the traditional metrics (such as MOTA, IDF1), can reflect the comprehensive ability of the tracker more comprehensively, and is especially suitable for algorithm evaluation and optimization in the scenario of this paper.

The formulas for the evaluation metrics covered above are defined below:


$$IDF1=\frac{2\times IDTP}{2\times IDTP+IDFP+IDFN}$$ (11)

In this formula, IDTP represents the number of identity true positives, IDFP denotes the number of identity false positives, and IDFN indicates the number of identity false negatives.


$$MOTA=1-\frac{\sum {\mathrm{\backslash nolimits}}_{t}\left(FN_{t}+FP_{t}+IDS_{t}\right)}{\sum {\mathrm{\backslash nolimits}}_{t}GT_{t}}$$ (12)

Where $FN_{t}$ denotes the number of false negatives at frame *t*, $FP_{t}$ represents the number of false positives a*t* frame *t*, $IDS_{t}$ indicates the number of identity switches occurring at frame *t*, and $GT_{t}$ is *t*he number of ground truth objects at frame *t*.


$$IDS=\sum {\mathrm{\backslash nolimits}}_{t}IDS_{t}$$ (13)

Where $IDS_{t}$ indicates the number of identity switches occurring at frame *t.*


$$HOTA=\sqrt{DetA\times AssA}$$ (14)

Where DetA refers to Detection Accuracy, AssA denotes Association Accuracy.


$$DetA=\frac{TP}{TP+FP+FN}$$ (15)

Where TP denotes the number of true positives, FP refers to the number of false positives, FN represents the number of false negatives.


$$AssA=\frac{\sum {\mathrm{\backslash nolimits}}_{c\in TP}A(c)}{TP}$$ (16)

In this formula, A(c) denotes the association accuracy for each true positive detection *c*.

### Implementation details

The YOLOX detector, for the DanceTrack dataset, uses the YOLOX weights provided from DanceTrack. For the MOT17 dataset, the public weights provided by ByteTrack for the MOT17 test set were used, which were only used to generate proposals for all images before training MOTR-ConvNext. Referring to the design of MOTRv2, for all YOLOX prediction frames, as long as the confidence score exceeds 0.05, they are retained as proposals. Retaining the low confidence detection results is used to improve the proposal recall in dense scenes.

MOTR-ConvNext, MOTR-ConvNext is implemented based on Deformable-DETR, which uses the ConvNext-Base backbone network for feature extraction, and the entire MOTR-ConvNext model is trained on 2 GPUs, each with a batch size of 1. $\omega_{s}$ and $\omega_{l}$ are set to 0.4 and 0.6, respectively, in order to enhance trajectory continuity under frequent occlusions while maintaining short-term matching accuracy. Referring to the experimental design of MOTR, $\lambda_{cls}^{s}$, $\lambda_{l1}^{s}$, and $\lambda_{giou}^{s}$ are set to 2, 5, and 2, respectively. To provide enough expressive power to capture the temporal dynamics of the object while taking into account the computational efficiency, D is set to 256. For the DanceTrack experiments, YOLOX is followed and HSV enhancement for training. During the training process, following the ablation experiments of MOTR, the track queries are generated based on the detect query when the confidence of the detect query is higher than a threshold value of 0.5. This can effectively suppress low-confidence false positives while retaining enough high-confidence detect queries for generating reliable track queries. Too low a threshold (e.g., 0.3) introduces a large number of low confidence noise detections, leading to an increase in false initialization, while too high a threshold (e.g., 0.7) omits some of the small or occluded targets and reduces the initialization recall. To avoid overfitting in complex action scenarios, 5 epochs are trained with a fixed clip size of 5, the sampling step within the frame is randomly selected from 1 to 10, and the initial learning rate is set to $2\times 10^{-4}$, which is reduced by a factor of 10 at the 4^th^ epoch. For the MOT17 experiments, the number of training epochs is adjusted to 50 to fully adapt to the relatively stable pedestrian scenes, and the learning rate is reduced at the 40^th^ epoch.

For the TQMM module, the memory capacity M is set to 300 based on the maximum number of targets in the dataset and the GPU’s memory capacity, which can cover all the historical trajectories in the high-density scenario without causing GPU overflow. The number of history frames $T_{S}$ is set to 3 to ensure sufficient short-term timing context information to enhance the association stability while controlling the computational complexity. The window length $T_{W}$ is set to 100 to avoid the introduction of stale tracks by an excessively long window while sufficiently covering all common occlusion scenarios in the dataset. According to the ablation experiments, the cosine similarity threshold *S* is set to 0.6 to achieve the optimal balance between the association precision and recall rate, and the maximum number of disappearing frames threshold F is set to 60 to achieve the optimal trade-off between noise suppression and maintaining the robustness of the association in the long-term. The smoothing factor α is set to 0.9, which has a moderately fast bias response and is suitable for the highly dynamic scenario of the dataset in this paper, and allows the trajectory features to gradually adapt to appearance changes while ensuring stability.

### Comparison with state-of-the-art methods

In this section, we compare UMOT with previous state-of-the-art methods on the two MOT benchmarks mentioned above, i.e., DanceTrack and MOT17. The results of these two benchmarks are reported in Tables 1–2, respectively. The data for the other methods come from MOTR [3], MOTRv2 [4], and MOTRv3 [14].

**Table 1:** Comparison with existing methods on the DanceTrack dataset.

Method	HOTA↑^a^	AssA↑	DetA↑	MOTA↑	IDF1↑
**CNN-based**					
FairMOT [9]	39.7	23.8	66.7	82.2	40.8
ByteTrack [10]	47.7	32.1	71.0	89.6	53.9
OC-SORT [11]	55.1	38.3	80.3	92.0	54.6
**Transformer-based**					
TransTrack [15]	45.5	27.5	75.9	88.4	45.2
MOTR [3]	54.2	40.2	73.5	79.7	51.5
MOTRv2 [4]	69.9	59.0	83.0	91.9	71.7
MOTRv3 [14]	70.4	59.3	83.8	92.9	72.3
**UMOT(OURS)**	**70.3**	**60.1**	**83.4**	**91.4**	**73.8**

^a^↑indicates higher scores are better.

**Table 2:** Comparison with existing methods on the MOT17 dataset.

Method	HOTA↑^a^	AssA↑	DetA↑	MOTA↑	IDF1↑	IDS ↓^b^
**CNN-based**						
FairMOT [9]	59.3	58.0	60.9	73.7	72.3	3303
ByteTrack [10]	63.1	62.0	64.5	80.3	77.3	2196
OC-SORT [11]	63.2	63.2	/	78.0	77.5	/
**Transformer-based**						
TransTrack [15]	54.1	47.9	61.6	74.5	63.9	3663
MOTR [3]	57.8	55.7	60.3	73.4	68.6	2439
MOTRv3 [14]	60.2	58.7	62.1	75.9	72.4	2403
MOTRv2 [4]	62.0	60.6	63.8	78.6	75.0	/
**UMOT(OURS)**	**62.9**	**61.8**	**64.4**	**77.8**	**77.2**	**1807**

^a^↑ indicates higher scores are better.

^b^↓ indicates lower scores are better.

Table 1 shows the performance of UMOT compared to other SOTA methods on the DanceTrack test set. Notably, IDS is not reported in the DanceTrack evaluation. This is because the dataset focuses on identity consistency measured by IDF1, while IDS fluctuates greatly in scenarios with highly similar appearances and frequent occlusions, making it less indicative of tracking performance. Moreover, this metric is not provided by the official evaluation toolkit or reported in mainstream benchmark papers.

From the Table 1, it can be concluded that the IDF1 of UMOT is improved by 1.5 compared to MOTRv3 (72.3) and 2.1 compared to MOTRv2 (71.7), which verifies the validity of the long-term association module. The TQMM module in UMOT dynamically maintains the memory bank through EMA smoothing update and sliding window cleanup, and when the target is reproduced, it is dynamically maintained through the Hb module (Historical backtracking Module) to restore its identity. The AssA of UMOT (60.1) is higher than that of MOTRv3 (59.3), suggesting that its trajectories are more robust to ID consistency in time. We attribute this to the hierarchical features of the ConvNeXt backbone network enhancing the modelling of complex motions, combined with the Temporal and Spatial Enhancement Module (TEM) of the TQMM, which optimizes trajectory continuity through multi-frame attentional fusion. UMOT’s HOTA is 0.4 higher than MOTRv2 and slightly lower than MOTRv3 by 0.1, but its association accuracy (60.1) significantly outperforms that of MOTRv3 (59.3) and MOTRv2 (59.0). For the combined detection (DetA) and association (AssA) performance of HOTA, UMOT’s long-term association module (TQMM+Hb) reduces ID switching through historical trajectory recovery (manifested as an IDF1 enhancement), but the introduced trajectory noise leads to a slight decrease in DetA, which in turn leads to a slight decrease in HOTA. The MOTA of UMOT (91.2) is slightly lower than that of MOTRv2 (91.9), and we hypothesize that the indicator MOTA is more focused on detection and short-term tracking and will ignore the continuity of the trajectory, whereas UMOT has a greater advantage in long-term correlation, leading to its slightly lower MOTA.

Table 2 demonstrates the performance comparison between UMOT and other SOTA methods on the MOT17 test set.

From the Table 2, it can be concluded that UMOT outperforms MOTRv3, i.e., the HOTA is improved by 2.7 and the IDF1 is improved by 4.8. The IDS of UMOT is 33% lower than that of MOTRv3, which indicates that the trajectories obtained are continuous and robust. UMOT also performs better compared to MOTRv2. However, similar to the results in the DanceTrack test set, the MOTA of UMOT is still slightly lower than that of MOTRv2. We still surmise that due to the large advantage of UMOT in long-term correlation, it makes it slightly lower in MOTA, which is later verified by our ablation experiments. In addition, it can be seen that CNN-based methods (e.g., ByteTrack and OC-SORT) perform well in the MOT17 dataset, although they do not perform as well as UMOT in DanceTrack. We infer that this is due to the simplicity of the target trajectories in MOT17, and therefore by combining a powerful target detector, such as YOLOX, with a designed post-processing rule, they can track the targets in MOT17 well.

Although the improvement in metrics such as IDF1 on the DanceTrack dataset is relatively small (e.g., a 1.5% increase in IDF1 compared to MOTRv3 in Table 1), such improvements are significant in dense multi-object tracking scenarios, particularly in highly challenging and complex environments like DanceTrack. In such scenarios, there are often a large number of targets with highly similar appearances and frequent occlusions, requiring the model to accurately match and maintain target identities within extremely short time frames, leaving little room for error. In this context, even a 1%−2% performance improvement often indicates significant optimizations in critical aspects such as occlusion handling, identity switching control, and trajectory recovery. Therefore, we believe that the stable improvements demonstrated by UMOT in the DanceTrack test fully reflect its progress in target tracking robustness and identity consistency.

Additionally, the MOT17 dataset is less complex and challenging compared to DanceTrack. UMOT’s strong performance on this dataset further validates the aforementioned conclusions. Specifically, compared to MOTRv2 and MOTRv3, UMOT achieved improvements of 2.2% and 4.8% in the IDF1 metric, 0.9% and 2.7% in the HOTA metric. These results indicate that UMOT not only demonstrates stronger target recognition capabilities and trajectory continuity in high-density crowded scenes but also exhibits excellent cross-dataset robustness and generalization capabilities, further validating the effectiveness and practicality of its design modules across various application scenarios.

### Tracking performance under different target densities

To further analyze the tracking performance of different algorithms under varying numbers of targets, this experiment uses the DanceTrack validation set to group video sequences based on the number of targets and evaluates the tracking performance differences between UMOT and other advanced algorithms under different target densities.

The DanceTrack dataset features a consistent number of targets per frame across video sequences, allowing for more accurate grouping by average target count and reducing bias from frame-level fluctuations. Designed for complex motion patterns, high appearance similarity, and dense multi-object distributions, the DanceTrack dataset facilitates the evaluation of target identity preservation and long-term association robustness in crowded scenarios. These characteristics closely align with the objectives of this study. In contrast, the MOT17 dataset exhibits significant fluctuations in target count within sequences and primarily covers low to medium-density pedestrian scenes, lacking the dynamic complexity and visual challenges found in DanceTrack.

Given limited experimental resources, this study focuses on improving and analyzing the transformer architecture. The overall performance of UMOT relative to methods such as FairMOT, OC-SORT, and TransTrack has already been systematically evaluated in the previous section. Therefore, this experiment concentrates on transformer-based methods structurally similar to UMOT (i.e., MOTR and MOTRv2) to validate the effectiveness of the proposed TQMM and related modules under varying target density conditions. MOTRv3 is excluded due to the unavailability of its official implementation and pre-trained models at the time of our experiments. In the experiment, the metric values for each target quantity group are the average of all metric values across all video sequences within that group. Averaging metric values across all sequences within each group provides a balanced evaluation that mitigates the influence of video length or target count imbalance, thereby ensuring fair comparison across target density levels. Table 3 demonstrates the performance comparison under different target density groups.

**Table 3:** Performance comparison under different target density groups.

Group	Method	HOTA↑^a^	AssA↑	DetA↑	MOTA↑	IDF1↑	IDS ↓^b^
1-5	MOTR	50.6	32.4	79.8	85.3	47.2	25
	MOTRv2	70.4	58.6	88.8	94.4	69.1	10
	**UMOT(OURS)**	**78.3**	**69.5**	**93.5**	**98.4**	**80.2**	**7**
6-10	MOTR	57.3	44.2	76.0	79.3	56.5	42
	MOTRv2	70.9	60.7	85.0	93.4	73.4	23
	**UMOT(OURS)**	**77.9**	**70.6**	**89.3**	**95.5**	**85.2**	**18**
11-15	MOTR	43.3	34.1	58.5	61.2	43.6	72
	MOTRv2	54.8	44.0	71.1	80.7	57.2	41
	**UMOT(OURS)**	**61.8**	**52.7**	**76.0**	**86.4**	**69.7**	**30**
>15	MOTR	42.5	30.4	61.0	63.7	43.7	132
	MOTRv2	54.3	41.0	73.7	83.8	57.5	61
	**UMOT(OURS)**	**60.0**	**48.7**	**76.8**	**88.3**	**67.3**	**49**

^a^↑ indicates higher scores are better.

^b^↓ indicates lower scores are better.

As shown in Table 3, the tracking performance of all evaluated methods declines as the number of targets increases, underscoring the increasing difficulty of maintaining accurate detection and association in crowded scenarios. Among the compared methods, MOTR demonstrates the weakest performance across all density groups, particularly in the group with more than 15 targets, where HOTA and IDF1 drop to 42.5 and 43.7, respectively. The high number of identity switches (132) in this group further highlights MOTR’s limited robustness in handling heavy occlusion and complex interactions.

MOTRv2 shows notable improvements over MOTR, primarily due to enhanced detection recall. Compared to MOTR, it achieves average performance gains of 13% in HOTA and 17% in IDF1 across different density levels. However, its effectiveness in high-density scenarios remains inadequate. For example, in the group with more than 15 targets, the IDF1 drops to 57.5, and there are still 61 identity switches, indicating insufficient capacity for preserving long-term identity consistency under crowded conditions.

In contrast, UMOT consistently outperforms both baselines across all target density groups. In low-density settings (1–5 targets), UMOT achieves a HOTA of 78.3 and an IDF1 of 80.2, with only 7 identity switches, demonstrating near-optimal tracking performance. In medium-density scenarios (6–10 targets), UMOT achieves an IDF1 score of 85.2, outperforming MOTRv2 by 11.8%, which confirms the effectiveness of the proposed TQMM and other modules in enhancing identity consistency through temporal context and memory-based matching. Under high-density conditions (11–15 targets and more than 15 targets), UMOT maintains a clear performance advantage. Specifically, in the group with more than 15 targets, its HOTA is 60.0, representing improvements of 17.5% and 5.7% over MOTR and MOTRv2, respectively. Its IDF1 value is 67.3, representing improvements of 23.6% and 9.5% over MOTR and MOTRv2, respectively, and the number of identity switches has been reduced to 49. Moreover, UMOT consistently achieves the highest AssA and DetA scores across all density groups, maintaining an AssA score above 48 even in the most crowded scenes. This demonstrates its superior ability in long-term association and effective recovery from occlusions.

These results collectively demonstrate that UMOT exhibits strong robustness and scalability across varying levels of target density. In particular, it significantly reduces identity switches and tracking drift in crowded scenes, effectively addressing the limitations of existing transformer-based methods and confirming its potential for real-world applications in complex environments.

### Ablation study

In this section, we explore the effect of the component modules ConvNeXt, TQMM, and Historical backtracking Module (Hb) on the DanceTrack validation set, in addition to the effect of the maximum disappearing frames threshold and the similarity threshold in the TQMM component on the DanceTrack validation set. The comparison of different components on the DanceTrack validation set is shown in Table 4.

**Table 4:** Comparison of different components in the DanceTrack validation set.

	Components			Metrics					
Method	TQMM	ConvNeXt	Hb	HOTA↑^a^	AssA↑	DetA↑	MOTA↑	IDF1↑	IDS ↓^b^
1 Base				61.0	45.3	85.1	94.0	60.3	1590
2		√		63.2	48.1	85.6	95.1	63.4	1517
3	√		√	69.4	59.9	82.3	91.1	73.5	762
**4 UMOT**	**√**	**√**	**√**	**70.7**	**60.6**	**84.2**	**94.0**	**75.2**	**616**

^a^↑ indicates higher scores are better.

^b^↓ indicates lower scores are better.

For the ConvNeXt backbone network, ConvNeXt’s large receptive field (7 × 7 depth-separable convolution) enhances the ability to capture contextual information around the target and assists in localizing the occluded target in occluded scenarios, thereby optimizing detection and short-term motion prediction, which in turn improves the association accuracy (AssA) and reduces the number of identity switches (IDS).The hierarchical feature design of ConvNeXt complements the Transformer decoder to enhance the robustness of short-term association. We can see by comparing Method 1 with Method 2 that using ConvNeXt as the backbone network, HOTA improves from 61.0 to 63.2 (+2.2), AssA improves from 45.3 to 48.1 (+2.8), IDF1 improves from 60.3 to 63.4 (+3.1), and IDS decreases from 1590 to 1517 (−4.6%). However, DetA is only improved by 0.5, indicating that ConvNeXt has limited optimization of detection accuracy, further suggesting that it focuses more on associative feature extraction rather than detection accuracy improvement, and that it also has limited enhancement of metrics such as HOTA and AssA, and relies on synergy with other modules.

For the TQMM module and the History backtracking Module (Hb), the TQMM is responsible for storing and updating the historical track queries information, and the stored trajectory information includes the appearance features, motion states and timestamps, which provides rich priors for the subsequent cross-frame matching. For example, after a target disappears due to occlusion, the track queries retained by TQMM can quickly trigger associations when it reappears. Its dynamic memory management mechanisms (e.g., EMA and sliding window cleanup) are used to maintain the timeliness of trajectories and reduce redundant matches. And Hb utilizes the memory track queries stored in TQMM to perform correlation computation with the current unmatched detections to recover lost targets, effectively reducing the number of identity switches (IDS) and improving the IDF1 score. We can see by comparing Method 1 with Method 3 that adding the TQMM module and the Hb module significantly improves HOTA from 61.0 to 69.4 (+8.4), AssA from 45.3 to 59.16 (+14.6), IDF1 from 60.3 to 73.5 (+13.2), and IDS from 1590 to 760 (−52%). DetA, on the other hand, decreased from 85.1 to 82.3, probably due to the noise introduced by long-term associations interfering with the detection accuracy. For example, outdated trajectories in the memory bank may mislead the bounding box regression for the current frame. In addition, we also see a decrease in MOTA from 94.0 to 91.1, illustrating the subtle mutual exclusivity between the long-term correlation ability of the TQMM and Hb modules and the MOTA metric, thus confirming our speculations in the comparison experiments.

In terms of overall structure, the synergistic ability of the three modules is able to compensate for the shortcomings of each other’s modules. The global features of ConvNeXt provide robust initial states of trajectories for the TQMM and Hb module, whereas the long-term correlation of the TQMM and Hb module compensates for the shortcomings of ConvNeXt in time series modelling. We can see by comparing Method 2 with Method 4 that after adding the TQMM module and the Hb module to ConvNeXt as the backbone network, HOTA improves from 63.2 to 70.7 (+7.5), AssA improves from 48.1 to 60.6 (+12.5), and IDF1 improves from 63.4 to 75.2 (+12.2), indicating that the TQMM module and Hb module have improved the long-term correlation metrics of the model for the ConvNeXt backbone network. By comparing Method 3 with Method 4, it can be seen that with the addition of ConvNeXt as the backbone network to the model of Method 3, the DetA improves from 82.3 to 84.2, and the metrics such as HOTA and AssA are slightly improved, suggesting that the improvement in the quality of the features of ConvNeXt’s backbone network offsets some of the noise interference introduced by TQMM module and Hb module.

**Fig 6:**
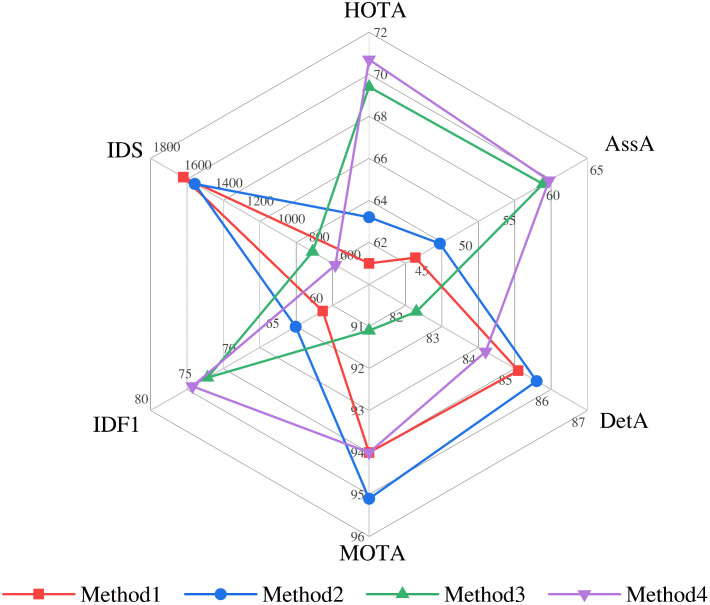
Comparison chart of performance of different components. Description of metrics: HOTA is Higher Order Tracking Accuracy, AssA is Association Accuracy, DetA is Detection Accuracy, MOTA is Multi-Objective Tracking Accuracy, IDF1 is Identity F1 Score, and IDS is Number of Identity Switches.

In order to facilitate a more intuitive representation of the above performance analyses, we plot the performance comparison of different components on the DanceTrack validation set, as shown in Fig 6.

Through the above analysis, the ConvNeXt backbone network optimizes the independent performance of detection and motion prediction through large receptive field context modelling, hierarchical feature fusion and lightweight design, provides occlusion-resistant and low-noise feature representations for the long-term association module, and then achieves a balance between detection accuracy and identity preservation in complex scenarios through module synergy. TQMM and Hb achieve a seamless integration of long-term and short-term association in multi-object tracking through dynamic memory management. TQMM acts as a spatio-temporal repository of trajectory information to ensure the continuity and timeliness of the historical state, while Hb recovers the lost targets through correlation computation to significantly reduce the IDS and improve the IDF1. The synergistic optimization of the two with ConvNeXt enables UMOT to achieve SOTA performance in complex scenarios (e.g., dense occlusion, nonlinear motion).

The cosine similarity threshold (*S*) in this study is used to determine whether the target in the current frame belongs to the old target already stored in the TQMM. If the similarity is greater than or equal to the threshold, the target is regarded as an old target (tracked target), and its trajectory information is updated by EMA smoothing. If the similarity is less than the threshold, the target is regarded as a new target (nascent target), and the new trajectory is initialized and stored in the TQMM.

We chose the DanceTrack dataset for the ablation studies of similarity threshold because this dataset is specifically designed for evaluating the performance of multi-object tracking algorithms in complex scenarios with complex motion patterns, high target appearance similarity, dense target distribution, and frequent occlusions. In contrast, MOT17 focuses mainly on pedestrian tracking in urban scenes, where targets usually exhibit linear and predictable motion trajectories with less variation in appearance and fewer cases of severe occlusion, and thus the similarity threshold does not have as significant an impact on the tracking performance of MOT17 as it does in DanceTrack. It is important to note that the optimal cosine similarity threshold may vary from dataset to dataset, with different datasets differing in target density, motion patterns, and appearance similarity, which can affect the selection of the optimal threshold. Therefore, when applying the method to other datasets, it is recommended to recalibrate the similarity thresholds according to their specific characteristics.

Table 5 demonstrates the effect of different similarity thresholds on the performance of the UMOT framework on the DanceTrack validation set.

**Table 5:** Ablation studies of similarity threshold.

Similarity threshold (*S*)	HOTA↑^a^	AssA↑	MOTA↑	IDF1↑	IDS ↓^b^
0.5	69.7	59.1	93.9	73.7	656
**0.6**	**70.7**	**60.6**	**94.0**	**75.2**	**616**
0.7	70.6	60.6	93.9	75.1	609
0.8	68.7	58.1	91.9	72.8	725
0.9	64.9	53.8	87.6	68.2	775

^a^↑ indicates higher scores are better.

^b^↓ indicates lower scores are better.

For the cosine similarity threshold, if a low threshold is adopted, it will relax the conditions for the determination of old targets, which may introduce noisy trajectories (mistakenly treating the new target as the old target), leading to identity switches and trajectory contamination. If a high threshold is used, the determination of old targets will be strictly limited, and the old target that should be associated may be missed (mistakenly considering the old target as the new target), leading to trajectory breakage and redundant initialization.

As can be seen from the table, when the threshold is 0.5, IDS increases by 6.5% to 656 and HOTA decreases by 1.0 to 69.7 compared to the threshold 0.6. This suggests that the low threshold can lead to false association problems, such as mis-matching of similar-looking targets in dense scenarios (e.g., trajectory confusion in the case of dancers’ fast cross-movements). The false association further interferes with the stability of the bounding box regression and the accuracy of the motion prediction, leading to a significant degradation of association accuracy (AssA) and overall performance (HOTA). At a threshold of 0.8, IDF1 decreases by 2.4 to 72.8 and MOTA decreases by 2.1 to 91.9 compared to a threshold of 0.6. This suggests that too high threshold hinders the recovery of old targets, e.g., targets reproduced after a long period of occlusion can not be associated with historical trajectories and are forced to initialize redundant IDs.

When the threshold is 0.6, the HOTA reaches the peak (70.7), and the IDS reaches 616, indicating that at this time, the detection and association are synergistically optimized to effectively balance the mis-match and miss-match in the target determination. When the threshold is 0.7, each index is similar to the threshold of 0.6, which indicates that the threshold of 0.6–0.7 performs stably in DanceTrack, and can be used as a general configuration, and this paper chooses the similarity threshold of 0.6.

The maximum disappearing frames threshold defines the upper limit of the survival duration of the memory track queries in the TQMM module, i.e., the maximum number of frames that the target remains in the memory bank without being updated. The threshold setting directly affects the robustness of long-term association and the efficiency of memory management.

Consistent with the reasons mentioned above for the ablation studies of the similarity threshold, we chose to perform ablation studies on the maximum disappearing frames threshold using the DanceTrack dataset. This dataset possesses complex motion patterns, high target appearance similarity, dense target distributions, and frequent occlusions, and can comprehensively evaluate the performance of the proposed method under the most challenging scenarios. It should be noted that when applying the method to other datasets, the relevant thresholds should be recalibrated according to their specific characteristics (e.g., target density, motion patterns, and appearance similarity) to ensure optimal tracking results.

Table 6 demonstrates the impact of different maximum disappearing frames thresholds (Disappearing Frames Threshold, F) on the performance of UMOT on the DanceTrack validation set (20 FPS).

**Table 6:** Ablation studies with maximum disappearing frames threshold.

Disappearing Frames Threshold (frames)	Corresponding Time (ms)	HOTA↑^a^	AssA↑	MOTA↑	IDF1↑	IDS ↓^b^
10	500	69.5	58.1	94.7	73.1	743
30	1500	70.0	59.2	94.5	74.5	660
**60**	**3000**	**70.7**	**60.6**	**94.0**	**75.2**	**616**
80	4000	70.5	60.4	93.7	75.6	566

^a^↑ indicates higher scores are better.

^b^↓ indicates lower scores are better.

For the maximum disappearing frames threshold, setting too short a survival duration leads to targets that cannot be recovered due to brief occlusions, increased missed matches, and frequent track deletions force the system to initialize redundant identities, which destroys identity consistency. For example, in the DanceTrack scenario, the target is briefly lost (<10 frames) due to fast rotation, but the threshold is too short resulting in the memory bank deleting the trajectory in advance, the target cannot be recovered. As shown in the table, when maximum disappearing frames threshold is selected as 10 frames, the HOTA is 69.5 (−1.2), and the IDF1 is 73.1 (−2.1) compared with the threshold of 60 frames. Setting too long a survival duration to retain the failure trajectory (e.g., mis-matched or long-term deactivated targets) leads to noise to interfere with detection and association. In addition, the long-term residency of the failed trajectory comes to pollute the memory bank and increase the ambiguity of cross-frame matching. As shown in the table, when maximum disappearing frames threshold is selected as 80 frames, compared with the threshold of 60 frames, the HOTA is 70.5 (−0.2), AssA is 60.4 (−0.2), and the IDF1 is 75.6 (+0.4), IDS is 566 (−8.1%), despite the decrease in IDS, the accumulation of noisy trajectories leads to a decrease in association accuracy (AssA) and a reduction in the overall performance of the tracking.

With the above ablation study, the maximum disappearing frames threshold (F) is selected as 60 frames, UMOT achieves an optimal balance between long-term association robustness and noise suppression, with HOTA (70.7) and IDF1 (75.2) significantly outperforming the other configurations.

### Visualization and analysis

#### Comparative analysis of the duplicate phenomenon.

The Duplicate phenomenon refers to the misassignment of multiple ID numbers to the same target, resulting in track redundancy (e.g., a pedestrian is recognized as multiple independent targets). This is usually caused by overlapping detection boxes, short-term occlusion, or abrupt changes in appearance.

**Fig 7:**
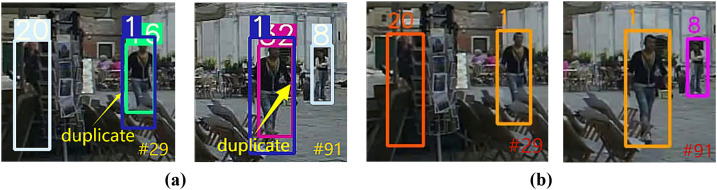
Comparison of Duplicate phenomenon results between UMOT and MOTR on MOT17 dataset. (a) Duplicate phenomenon of MOTR on MOT17 dataset (b) The comparison results of UMOT on the MOT17 dataset.

In MOTR, MOTR suffers from unstable bounding box regression due to detection-association task conflicts in the end-to-end framework, leading to the generation of multiple track queries for the same target, and the Duplicate phenomenon of MOTR on the MOT17 dataset is shown in Fig 7(a). While the TQMM module in UMOT suppresses the long-term accumulation of redundant trajectories by sliding window cleanup and EMA updates. The comparison results of UMOT on MOT17 dataset are shown in Fig 7(b).

As shown in Fig 7(a), we can see that a pedestrian is assigned two ID numbers 1 and 6 in frame 29 of MOTR, and the pedestrian is assigned two more ID numbers 1 and 32 in frame 91. From Fig 7(b) we can see that neither frame 29 nor frame 91 in UMOT shows that the pedestrian is assigned multiple ID numbers.

**Fig 8:**
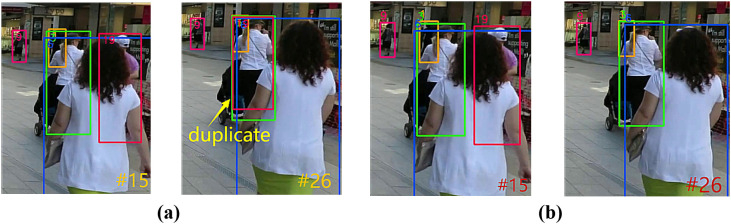
Comparison of Duplicate phenomenon results between UMOT and MOTRv2 on MOT17 dataset. (a) Duplicate phenomenon of MOTRv2 on the MOT17 dataset (b) The comparison results of UMOT on the MOT17 dataset.

**Fig 9:**
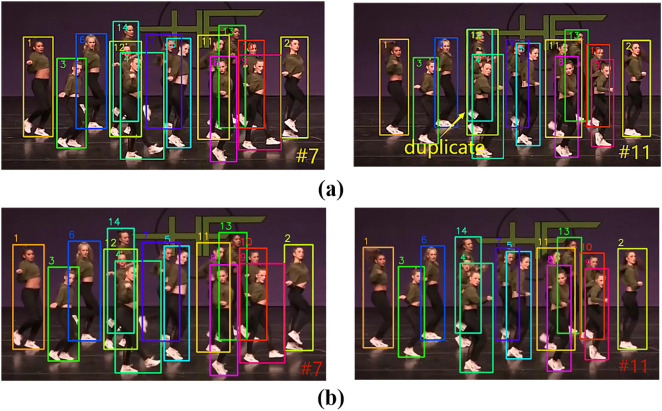
Comparison of Duplicate phenomenon results between UMOT and MOTRv2 on DanceTrack dataset. (a) Duplicate phenomenon of MOTRv2 on the DanceTrack dataset (b) The comparison results of UMOT on the DanceTrack dataset.

In MOTRv2, MOTRv2 mitigates the conflict by pre-training detector, but there are still duplicate trajectories in the dense crossover scenario. The Duplicate phenomenon of MOTRv2 on the MOT17 dataset is shown in Fig 8(a), and on the DanceTrack dataset is shown in Fig 9(a). The comparison results of UMOT on the MOT17 dataset are shown in 8(b) and those on the DanceTrack dataset are shown in Fig 9(b).

As shown in Fig 8(a), we can see that pedestrian 3 appears at the same time as pedestrian 19 in frame 15 of MOTRv2, but the original pedestrian 3 is assigned both ID numbers 3 and 19 in frame 26. And from Fig 8(b) we can see that the phenomenon does not occur in frame 26 in UMOT.

Similar to the case of Fig 8, from Fig 9(a) we can see that the original dancer 14 in MOTRv2 was assigned both ID numbers 12 and 14 in frame 11. And from Fig 9(b) we can see that the phenomenon does not occur in frame 11 in UMOT.

#### Comparative analysis of the id switch phenomenon.

ID Switch refers to the wrong switching of the target’s identity during tracking (e.g., ID swapping or ID change when dancers cross-move), which is mainly caused by occlusion, similarity in appearance, or sudden changes in motion.

**Fig 10:**
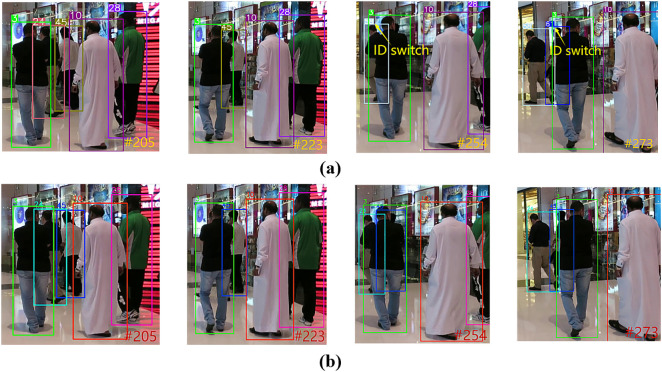
Comparison of ID switch phenomenon results between UMOT and MOTR on MOT17 set. (a) The phenomenon of ID switch for MOTR on the MOT17 dataset (b) The comparison results of UMOT on the MOT17 dataset.

**Fig 11:**
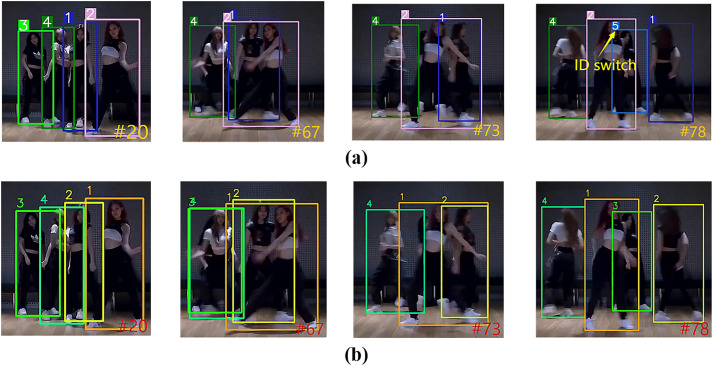
Comparison of ID switch phenomenon results between UMOT and MOTR on DanceTrack set. (a) The phenomenon of ID switch for MOTR on the DanceTrack dataset (b) The comparison results of UMOT on the DanceTrack dataset.

In MOTR, MOTR is unable to match the historical trajectories during target reproduction due to the lack of a long-term association mechanism, resulting in frequent ID switching. The ID Switch phenomenon of MOTR on the MOT17 dataset is shown in Fig 10(a), and that on the DanceTrack dataset is shown in Fig 11(a). UMOT computes the correlation between the unmatched detections and the memory track queries (storing up to 300 targets) through cross-frame correlation between the TQMM module and the Hb module to recover the targets that have been lost for a long period of time due to occlusion. The comparison results of UMOT on the MOT17 dataset are shown in Fig 10(b), and those on the DanceTrack dataset are shown in Fig 11(b).

As shown in Fig 10(a), the MOTR occludes pedestrian 24 at frame 223, and pedestrian 24 reappears at frame 254, but due to the lack of long-term association mechanism in the MOTR, it can not match the historical trajectories when the target is reproduced, and the ID of the original pedestrian 24 is changed from 24 to 58, and meanwhile, the original pedestrian 45 is occluded, and the ID of the original pedestrian 45 is changed from 45 to 61 when the original pedestrian 45 reappears at frame 273, while it can be clearly seen from Fig 10(b) that all the pedestrians in each frame of the UMOT have not changed their IDs.

Similar to the case of Fig 10, from Fig 11(a) and 11(b) we can see that after a few tens of frames of masking in MOTR, the ID of the original dancer 3 changes from 3 to 5 at frame 78, whereas the phenomenon does not occur at frame 67 in UMOT.

**Fig 12:**
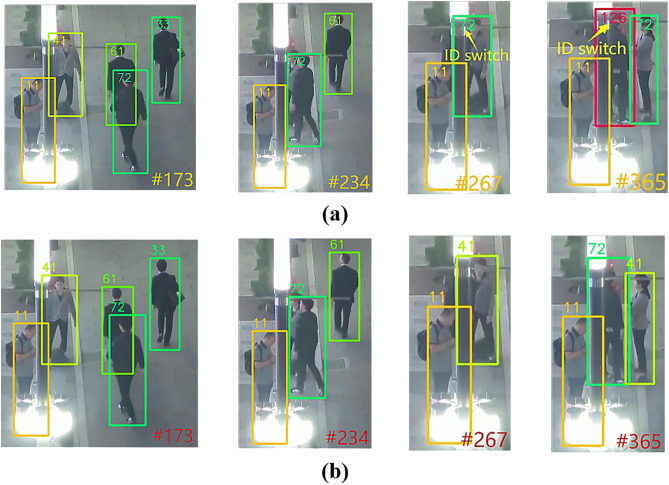
Comparison of ID switch phenomenon results between UMOT and MOTRv2 on MOT17 dataset. (a) ID switch phenomenon of MOTRv2 on MOT17 dataset (b) The comparison results of UMOT on the MOT17 dataset.

**Fig 13:**
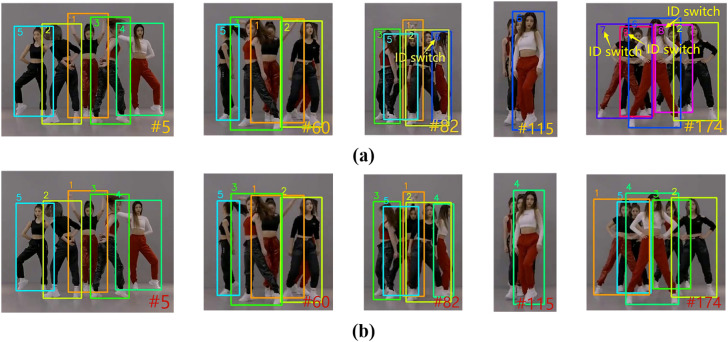
Comparison of ID switch phenomenon results between UMOT and MOTRv2 on DanceTrack dataset. (a) ID switch phenomenon of MOTRv2 on DanceTrack datase (b) The comparison results of UMOT on the DanceTrack dataset.

In MOTRv2, MOTRv2 mitigates some of the detection problems by detector bootstrapping, but still relies on short-term matching after long-term occlusions. The ID Switch phenomenon of MOTRv2 on the MOT17 dataset is shown in Fig 12(a), and on the DanceTrack dataset is shown in Fig 13(a). The comparison results of UMOT on the MOT17 dataset are shown in Fig 12(b) and those on the DanceTrack dataset are shown in Fig 13(b).

As shown in Fig 12(a), pedestrian 41 is occluded at frame 234 in MOTRv2, and pedestrian 41 reappears at frame 267, but due to MOTRv2’s reliance on short-term matching and the lack of long-term association, the ID of the original pedestrian 41 is changed from 41 to 72, and at the same time, the original pedestrian 72 is occluded, and the ID of the original pedestrian 72 is changed from 72 to 126 when the original pedestrian 72 reappears at frame 365, while it can be clearly seen from Fig 12(b) that all pedestrians in each frame of UMOT have not been changed.

As shown in Fig 13(a), dancer 41 is occluded at frame 60 in MOTRv2, and dancer 4 reappears at frame 60 with the ID of the original dancer 4 changing from 4 to 6. Occlusion occurs again at frame 115, and when all the dancers reappear at frame 174, the ID of the original dancer 1 is changed from 1 to 7, that of the original dancer 3 is changed from 2 to 8, and the ID of the original dancer 5 is changed from 5 to 9. And it is obvious from Fig 13(b) that the IDs of all dancers in each frame of UMOT remain unchanged.

Through the above visualization and analysis, we can get that UMOT significantly reduces the phenomena of Duplicate and ID Switch in complex scenarios (e.g., DanceTrack and MOT17) by using a unified framework of long- and short-term association, indicating that the model shows significant advantages in complex motion modelling and identity preservation.

### Efficiency analysis

To comprehensively evaluate the practical application value of the proposed UMOT method, in addition to verifying its advantages in tracking accuracy and robustness, it is also necessary to analyze its computational efficiency, model size, and resource consumption. This experiment aims to compare the inference speed, number of parameters, and model size of UMOT with those of current mainstream multi-object tracking algorithms (MOTR, MOTRv2, ByteTrack) to assess its feasibility and performance trade-offs in real-world deployment scenarios. It should be noted that MOTRv3 was not included in this efficiency comparison due to the unavailability of its official implementation and pre-trained model at the time of experimentation. The experiment was conducted on a Tesla V100 GPU (32GB) using the DanceTrack validation set for inference efficiency analysis. The results are presented in the Table 7.

**Table 7:** Comparison of inference speed and model size for different tracking algorithms.

Method	FPS	Parameters (M)	Model size (MB)
**CNN-based**			
FairMOT	8.3	34.4	247
ByteTrack (YOLOX-X^a^)	36	99.1	187
OC-SORT (YOLOX-X)	22.7	99.7	187
**Transformer-based**			
TransTrack	12.5	46.9	53.8
MOTR	7.5	43.9	169
MOTRV2 (YOLOX-S^b^)	8.9	50.7	187.2
**UMOT (OURS)**	**9**	**114.6**	**367.2**

^a^YOLOX-X indicates that this method uses YOLO-X as the detector.

^b^YOLOX-S indicates that this method uses YOLO-S as the detector.

As can be seen from the table, CNN-based methods generally outperform Transformer-based methods in terms of inference efficiency. Among them, ByteTrack achieves the highest 36 FPS, meeting real-time or even high-frame-rate inference requirements, while OC-SORT reaches 22.7 FPS, suitable for most practical applications. FairMOT, which simultaneously performs detection and ReID feature extraction, has a relatively low inference speed of only 8.3 FPS.

Among Transformer-based methods, TransTrack achieves an inference speed of 12.5 FPS, primarily due to its use of the lightweight DLA-34 as the backbone. MOTR and MOTRv2, which adopt the Transformer encoder-decoder architecture, achieve further reductions in inference speed to 7.5 FPS and 8.9 FPS, respectively, indicating that the Transformer decoder incurs significant computational overhead in dense scenes, thereby limiting the model’s real-time performance.

UMOT achieves an inference speed of 9.0 FPS, slightly higher than MOTR and MOTRv2, indicating that the proposed long-term and short-term correlation modules enhance tracking performance while maintaining acceptable inference efficiency among Transformer-based trackers. However, compared to TransTrack and all CNN-based methods, UMOT still lags behind in inference speed. Additionally, UMOT has the highest parameter count at 114.6M and a model size of 367.2 MB, significantly higher than MOTR (169 MB) and MOTRv2 (187.2 MB). This is primarily due to UMOT’s design of additional attention layers and memory units on top of the transformer encoder-decoder architecture, which enhances tracking robustness and identity preservation in dense scenes but also results in higher computational complexity and memory consumption.

In summary, UMOT demonstrates the optimal trade-off between tracking accuracy and robustness among transformer-based trackers, with slightly better inference efficiency than the MOTR series, proving the effectiveness of long-term and short-term correlation modules in significantly improving tracking performance while maintaining acceptable computational complexity. Although UMOT lags behind CNN-based methods in inference speed and model compactness, this is an expected trade-off. CNN-based trackers are inherently more lightweight than Transformer-based architectures, which are designed to capture richer temporal-spatial dependencies. Future work can further reduce computational complexity and parameter count through methods such as backbone network lightweighting, module pruning, memory compression, and model distillation, while maintaining tracking robustness in dense scenes and improving model deployability and real-time performance.

## Discussion

In this paper, a unified framework for long- and short-term association for multi-object tracking, UMOT, is proposed for the problem of trajectory breakage caused by long-term occlusions in multi-object tracking. Through the short-term association module, this paper systematically integrates the pre-trained YOLOX detector with the MOTR-ConvNext network to achieve efficient modelling of the target’s motion and appearance characteristics in adjacent frames. At the same time, using the Track Queries Memory Module and the Historical backtracking Module, the smooth update and cross-frame correlation mechanism of the historical trajectory information is successfully constructed, which effectively recovers the target information that has been lost for a long time.

In the UMOT framework, each component within the short-term and long-term modules plays an essential role in achieving robust multi-object tracking. The short-term correlation module combines a pre-trained YOLOX detector, which provides reliable detection proposals and positional priors, with the MOTR-ConvNext model that enhances feature representation through its expanded receptive field and hierarchical feature fusion. This combination allows the model to jointly model motion and appearance features, thereby improving short-term association accuracy and reducing ID switches. The long-term correlation module includes the Track Query Memory Module (TQMM) for storing historical trajectory features to support re-identification of long-lost targets, the Temporal-Spatial Enhancement Module (TEM) for aggregating contextual information across frames to strengthen trajectory consistency, and the Exponential Moving Average (EMA) updating strategy to smoothly update feature representations and maintain stability under appearance variations. Together, these components ensure that UMOT effectively addresses both short-term motion prediction and long-term identity preservation, resulting in enhanced tracking robustness and performance in complex, dense scenarios.

Extensive experiments demonstrate that UMOT achieves superior tracking accuracy and long-term identity preservation compared to existing Transformer-based methods, particularly in high-density and crowded scenarios. This performance gain stems from the integration of memory-enhanced long-term association mechanisms and ConvNeXt-based feature extraction, which together strengthen temporal consistency and appearance modeling. While UMOT incurs a relatively higher computational load than CNN-based trackers due to its attention modules and memory structures, it maintains a reasonable efficiency-performance trade-off among Transformer-based approaches and remains feasible for most practical applications.

In future work, we will focus on optimizing model size and inference efficiency through backbone lightweighting, module pruning, and memory compression to enhance its deployability on resource-constrained edge devices while maintaining high tracking robustness. Moreover, we also plan to incorporate standard cross-validation-based statistical analyses to further strengthen the generalizability and significance of the proposed method’s performance gains across different datasets and scenarios.

## Supporting information

S1 File **S1 Fig.** Visualisation of the ablation studies of similarity threshold. **S2 Fig.** Visualisation of the ablation studies of the maximum disappearing frames threshold. **S1 Table.** Complete raw comparison results of different components in the DanceTrack validation set. **S2 Table.** Complete raw results of the ablation studies of similarity threshold. S**3 Table.** Complete raw results of the ablation studies of the maximum disappearing frames threshold. **S4 Table.** Complete raw results of the tracking performance under different target densities. **S1 Video.** Demonstration of tracking results for UMOT on the MOT17 dataset. **S2 Video.** Demonstration of tracking results for UMOT on the DanceTrack dataset. **S1 Video Caption.** S1 video documentation. **S2 Video Caption.** S2 video documentation.
(ZIP)
