# Supplementary material for: UMOT: A unified framework for long- and short-term association for multi-object tracking
Source: PLoS One. 2025 Sep 26;20(9):e0332709. doi: 10.1371/journal.pone.0332709 (PMC12469223; doi:10.1371/journal.pone.0332709)
Supplement: S1 File — S1 Fig. Visualisation of the ablation studies of similarity threshold. S2 Fig. Visualisation of the ablation studies of the maximum disappearing frames threshold. S1 Table. Complete raw comparison results of different components in the DanceTrack validation set. S2 Table. Complete raw results of the ablation studies of similarity threshold. S3 Table. Complete raw results of the ablation studies of the maximum disappearing frames threshold. S4 Table. Complete raw results of the tracking performance under different target densities. S1 Video. Demonstration of tracking results for UMOT on the MOT17 dataset. S2 Video. Demonstration of tracking results for UMOT on the DanceTrack dataset. S1 Video Caption. S1 video documentation. S2 Video Caption. S2 video documentation. (ZIP) [file pone.0332709.s001.zip › Supporting_Information/S1_Video_Caption.docx]

S1 Video. ****Demonstration of tracking results for UMOT on the MOT17 dataset.****

Resolution: 1920×1080 (1080p)

Frame rate: 30 fps

Format: MP4 (H.264 encoding)

Copyright: © Yongxing Ke, 2025. Licensed under CC-BY 4.0.

Content Summary:

Real-time tracking results of UMOT on the MOT17 dataset, showcasing robust performance in scenarios with occlusions and identity preservation.

Key features demonstrated: High-density crowd handling, trajectory recovery for long-term lost targets.

Associated Citation: Refer to "Experiments" section and Supporting Information (S1 Video).
