# Supplementary material for: UMOT: A unified framework for long- and short-term association for multi-object tracking
Source: PLoS One. 2025 Sep 26;20(9):e0332709. doi: 10.1371/journal.pone.0332709 (PMC12469223; doi:10.1371/journal.pone.0332709)
Supplement: S1 File — S1 Fig. Visualisation of the ablation studies of similarity threshold. S2 Fig. Visualisation of the ablation studies of the maximum disappearing frames threshold. S1 Table. Complete raw comparison results of different components in the DanceTrack validation set. S2 Table. Complete raw results of the ablation studies of similarity threshold. S3 Table. Complete raw results of the ablation studies of the maximum disappearing frames threshold. S4 Table. Complete raw results of the tracking performance under different target densities. S1 Video. Demonstration of tracking results for UMOT on the MOT17 dataset. S2 Video. Demonstration of tracking results for UMOT on the DanceTrack dataset. S1 Video Caption. S1 video documentation. S2 Video Caption. S2 video documentation. (ZIP) [file pone.0332709.s001.zip › Supporting_Information/S2_Video_Caption.docx]

**S2 Video. **Demonstration of tracking results for UMOT on the DanceTrack dataset.****

**Resolution:** 1920×1080 (1080p)

**Frame rate:** 30 fps

**Format:** MP4 (H.264 encoding)

**Copyright:** © Yongxing Ke, 2025. Licensed under CC-BY 4.0.

**Content Summary:**

Ttracking results of UMOT on DanceTrack dataset, highlighting effectiveness in complex motion and occlusion scenarios.

**Key features demonstrated:** Multi-target interaction in dance sequences, identity consistency under rapid motion.

**Associated Citation:** Refer to "Experiment" section and Supporting Information (S2 Video).
