# Supplementary figures and images for: UMOT: A unified framework for long- and short-term association for multi-object tracking
Source: PLoS One. 2025 Sep 26;20(9):e0332709. doi: 10.1371/journal.pone.0332709 (PMC12469223; doi:10.1371/journal.pone.0332709)

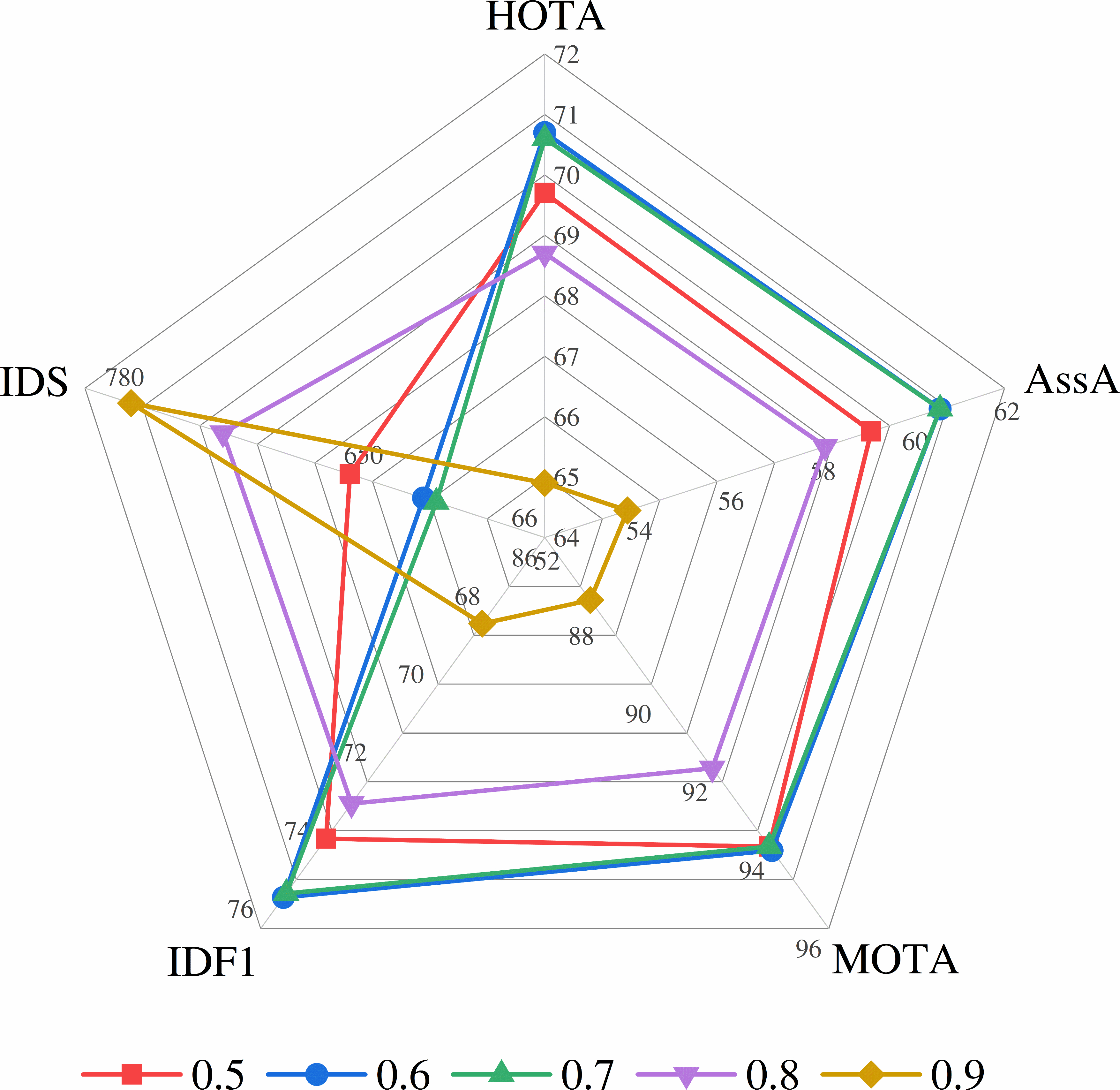

Supplement: S1 File — S1 Fig. Visualisation of the ablation studies of similarity threshold. S2 Fig. Visualisation of the ablation studies of the maximum disappearing frames threshold. S1 Table. Complete raw comparison results of different components in the DanceTrack validation set. S2 Table. Complete raw results of the ablation studies of similarity threshold. S3 Table. Complete raw results of the ablation studies of the maximum disappearing frames threshold. S4 Table. Complete raw results of the tracking performance under different target densities. S1 Video. Demonstration of tracking results for UMOT on the MOT17 dataset. S2 Video. Demonstration of tracking results for UMOT on the DanceTrack dataset. S1 Video Caption. S1 video documentation. S2 Video Caption. S2 video documentation. (ZIP) [file pone.0332709.s001.zip › Supporting_Information/S1_Fig.tif]

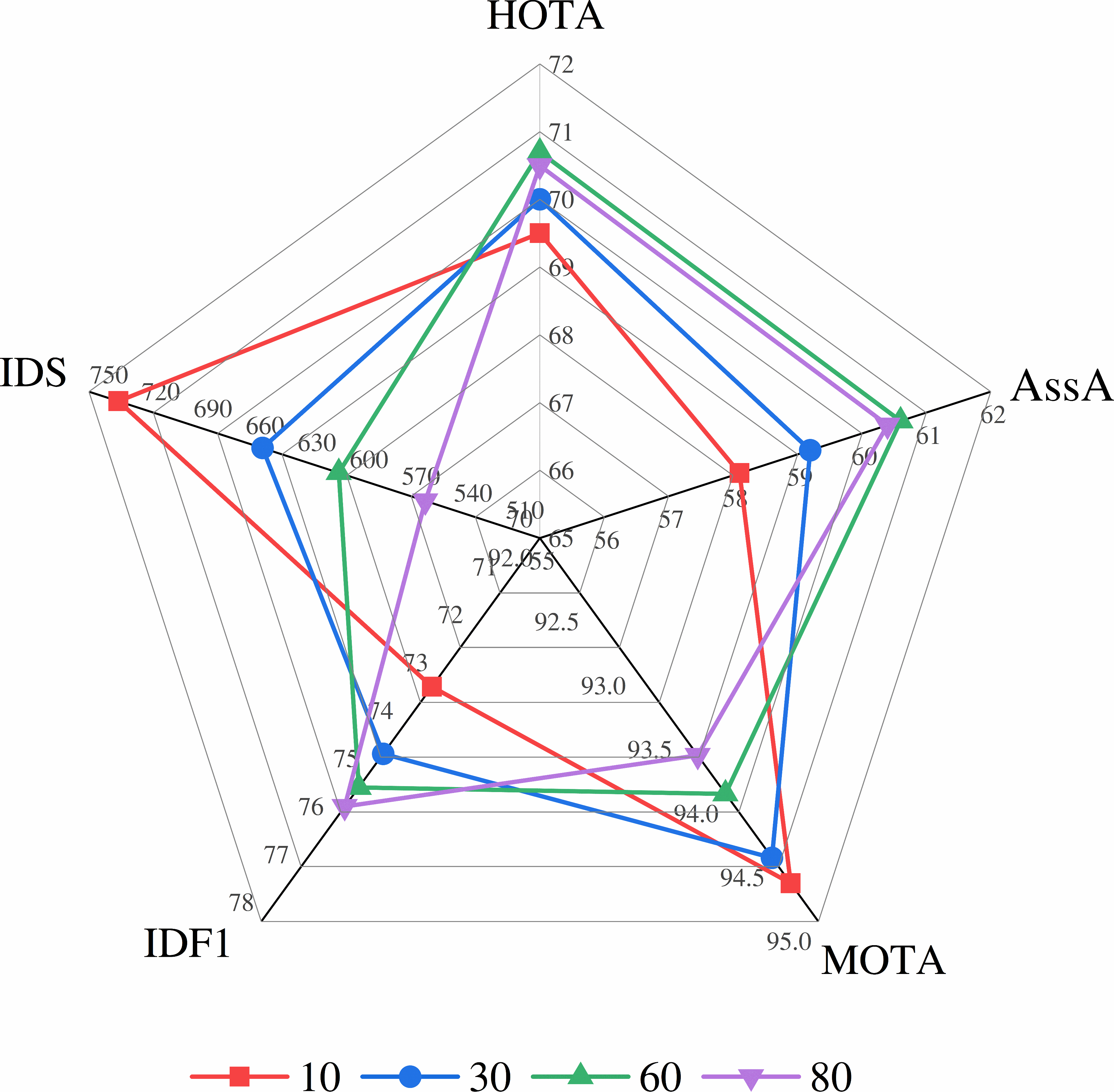

Supplement: S1 File — S1 Fig. Visualisation of the ablation studies of similarity threshold. S2 Fig. Visualisation of the ablation studies of the maximum disappearing frames threshold. S1 Table. Complete raw comparison results of different components in the DanceTrack validation set. S2 Table. Complete raw results of the ablation studies of similarity threshold. S3 Table. Complete raw results of the ablation studies of the maximum disappearing frames threshold. S4 Table. Complete raw results of the tracking performance under different target densities. S1 Video. Demonstration of tracking results for UMOT on the MOT17 dataset. S2 Video. Demonstration of tracking results for UMOT on the DanceTrack dataset. S1 Video Caption. S1 video documentation. S2 Video Caption. S2 video documentation. (ZIP) [file pone.0332709.s001.zip › Supporting_Information/S2_Fig.tif]
